# Impact of aging on primary liver cancer: epidemiology, pathogenesis and therapeutics

**DOI:** 10.18632/aging.203620

**Published:** 2021-10-11

**Authors:** Rocio I.R. Macias, Maria J. Monte, Maria A. Serrano, Jesús M. González-Santiago, Isabel Martín-Arribas, André L. Simão, Rui E. Castro, Javier González-Gallego, José L. Mauriz, Jose J.G. Marin

**Affiliations:** 1 Experimental Hepatology and Drug Targeting (HEVEPHARM) Group, University of Salamanca, IBSAL, Salamanca, Spain; 2 Department of Gastroenterology and Hepatology, University Hospital of Salamanca, IBSAL, Salamanca, Spain; 3 Research Institute for Medicines (iMed.ULisboa), Faculty of Pharmacy, Universidade de Lisboa, Lisbon, Portugal; 4 Institute of Biomedicine (IBIOMED), University of León, León, Spain; 5 Centro de Investigación Biomédica en Red de Enfermedades Hepáticas y Digestivas (CIBERehd), Carlos III National Institute of Health, Madrid, Spain

**Keywords:** aging, cholangiocarcinoma, fragility, hepatocarcinoma, senescence

## Abstract

Aging involves progressive physiological and metabolic reprogramming to adapt to gradual deterioration of organs and functions. This includes mechanisms of defense against pre-malignant transformations. Thus, certain tumors are more prone to appear in elderly patients. This is the case of the two most frequent types of primary liver cancer, i.e., hepatocellular carcinoma (HCC) and intrahepatic cholangiocarcinoma (iCCA). Accordingly, aging hallmarks, such as genomic instability, telomere attrition, epigenetic alterations, altered proteostasis, mitochondrial dysfunction, cellular senescence, exhaustion of stem cell niches, impaired intracellular communication, and deregulated nutrient sensing can play an important role in liver carcinogenesis in the elders. In addition, increased liver fragility determines a worse response to risk factors, which more frequently affect the aged population. This, together with the difficulty to carry out an early detection of HCC and iCCA, accounts for the late diagnosis of these tumors, which usually occurs in patients with approximately 60 and 70 years, respectively. Furthermore, there has been a considerable controversy on what treatment should be used in the management of HCC and iCCA in elderly patients. The consensus reached by numerous studies that have investigated the feasibility and safety of different curative and palliative therapeutic approaches in elders with liver tumors is that advanced age itself is not a contraindication for specific treatments, although the frequent presence of comorbidities in these individuals should be taken into consideration for their management.

## INTRODUCTION

During the last decades, there has been a marked increase in life expectancy in most developed countries. The consequence is that the population is aging in these geographical areas. For instance, in early 2018, about 20% of the total population in the European Union had more than 65 years. This proportion is expected to reach 28.5% in 2050 [1], and a similar demographic evolution is expected to occur in the US and other developed countries. It is well-known that elders have an increased risk of developing chronic diseases, including some cancers. Primary liver cancer is the sixth most common cancer worldwide, with 80% of cases being diagnosed among patients who are 70 years or older, and it is the third leading cause of cancer-related death worldwide according to the WHO. Approximately, 85-90% of these cancers are hepatocellular carcinoma (HCC), which is currently the third cancer-related cause of death in men worldwide, and its incidence is regularly increasing [2]. Besides, intrahepatic cholangiocarcinoma (iCCA) accounts for 10-15% of all primary liver cancers, and the number of newly diagnosed cases per year is also rising [3], although its incidence rate has important geographical variations, reflecting local differences in risk factors [4]. The increasing incidence of liver cancer is partially attributable to the increase in morbidities commonly associated with aging, such as diabetes mellitus, alcoholic liver disease, and non-alcoholic fatty liver disease [3].

Aging is characterized by cellular senescence caused by the shortening of telomeres in successive cell divisions, which leads to a halt in the proliferation of somatic cells. Several processes such as DNA damage, epigenetic alteration, oxidative stress, mitochondrial dysfunction, and alteration of metabolic pathways can contribute to the senescence of cells and tissues and, at the same time, account for higher risk of liver cancer development [5].

The main challenge in managing the growing number of elderly patients who have liver cancer is their frequent multimorbidity and hence the associated simultaneous use of several types of drugs that can result in drug-drug interactions interfering with cancer treatment. Besides, a decline in the functional reserve of several organs and the fact that the metabolism is often altered reduces the tolerance. Moreover, it can produce or aggravate adverse drug reactions [6]. Although a personalized treatment is always desirable, this is particularly required in the case of elderly patients. After evaluating their individual characteristics, the treatment must include proper monitoring in order to guarantee an adequate treatment intensity while preventing or minimizing the occurrence of adverse events and a deterioration of quality of life due to treatment [7].

The concept of elderly patients has changed over time, complicating the analysis of results published at different times. The most recent studies use as cut-off age of the elderly 75 or even 80 years, while some time ago patients more than 65 years old were included in the group of older patients and some guidelines still use this cut-off [8]. Despite these differences, most studies have shown that advanced age alone should not be a reason to dismiss the oncological evaluation for any treatment in the first place. Still, the treatment of elderly requires that, prior to designing the therapeutic strategy, oncologists carefully consider age-related comorbidities.

## Age-associated risk factors

The risk of CCA, either iCCA, distal (dCCA), or perihilar (pCCA) increases with advancing age, especially in Japan and Western countries where, without the specific risk factors of certain regions, patients present an average age at diagnosis of approximately 70, which is ten years older than the average age for patients first diagnosed with HCC [9, 10]. A recent study analyzing cancer mortality in elderly patients showed that liver cancer mortality rates were similar in most countries, with a peak of 60 per 100,000 or below for men and 25 per 100,000 or below for women in the 80-to-84 range of age. Exceptionally, these values were higher in Japan [11].

Clinical characteristics are different in elderly *versus* young HCC patients (Table 1). Elderly patients with HCC are mainly female, which has been associated with their longer life expectancy, while younger patients are predominantly male [12]. Hepatitis B virus (HBV) infection is the most frequent etiology in young HCC patients, probably because the transmission mainly occurs in the perinatal period. In contrast, the most prevalent causes of HCC in elderly patients are chronic infection with hepatitis C virus (HCV) and non-alcoholic steatohepatitis (NASH), which usually occurs later in life. The effects of alcohol consumption and HCV infection on the development of HCC appear to be stronger with advancing age, but also moderate alcohol consumption throughout life can cause HCC in the elderly [13]. In the upcoming years, due to the improvement of antiviral treatments, HCV infections are expected to decrease worldwide. In contrast, due to the predicted increased incidence of non-alcoholic fatty liver disease (NAFLD) -recently termed metabolic associated fatty liver disease (MAFLD)- predisposing for NASH, a significant growth in the number of cases of elderly patients with HCC and NASH can be predicted.

**Table 1:** Characteristics of hepatocellular carcinoma in elderly compared with younger patients.

**Characteristic**	**Elderly**	**Young**	**Ref.**
**Gender**	Female > Male	Male > Female	[12]
**Etiology**	HCV >NASH	HBV	[13]
**Comorbidities**	Cirrhosis, HCV	Cirrhosis, HBV	[29]
	Hypertension, diabetes, coronary disease, cerebral infarction		
**Tumors**	Few nodules, big size	Multiple nodules, small size	[30, 31]
	Well-differentiated	Poorly differentiated	
	Infrequent vessel invasion	Frequent vessel invasion	
**Liver fibrosis**	Severe	Moderate	

HBV, hepatitis B virus; HCV, hepatitis C virus; NASH, non-alcoholic steatohepatitis.

MAFLD refers to liver steatosis in addition to overweight or obesity, diabetes mellitus type 2 or metabolic dysregulation [14]. The number of studies in aging patients analyzing the relationship between MAFLD and primary liver cancer remains still limited and are main focused on HCC. An US study of cohorts across a 6-year period (2004 to 2009) showed a 9% annual increase on the number of NAFLD patients with HCC. NALFD-HCC patients were older than HCC patients with other underlying diseases (73.0 years *vs.* 66.0 years), with a shorter survival time and death was more often as consequence of this primary liver cancer [15]. Similarly, the increased incidence rate of HCC between 2003 and 2011 was associated with an elevated prevalence of NAFLD in Taiwanese patients older than 65 years [16]. In addition, in U.K., patients with NAFLD-associated HCC were older than those with other HCC etiologies (71.3 years vs 67.1 years), being liver tumors less often detected by clinical surveillance, although their survival was comparable [17]. It has been proposed a potential relationship between NAFLD and iCCA, which suggests a common pathogenesis with HCC [18, 19], however, no clear association between aging and MAFLD has already been established in this cancer.

The fact that women seem to be less prone to suffer from some liver pathologies, such as MAFLD, until post-menopausal ages, having a “lag period” when compared to male [20] can also contribute to the higher incidence in more advance age. Thus, a Taiwanese study concluded that NAFLD could constitute a possible risk factor associated to the upward trend in HCC incidence in elderly women [16]. Moreover, it has been described that depletion of cholesterol synthesis by *Cyp51* knock-out leads to HCC progression in aging female mice, indicating that sex-dependent metabolic reprogramming of cholesterol metabolism can predispose for hepatocarcinogenesis in aging females [21]. Recently, estradiol has been related to prognosis in non-surgical HCC patients leading to a better mean survival probability in women than men, but this effect is reduced after menopause. *In vitro* studies have demonstrated that estradiol is able to inhibit the proliferation of HCC cell lines [22]. Besides, it has been reported that post-menopausal hormone replacement therapy plays a protective role in HCC [23, 24]. Contrarily, high circulating levels of estradiol, commonly found in male iCCA patients, have been associated with an increased risk of this type of primary tumor in both men and women [25, 26]. Additionally, long-term oral contraceptive use and hysterectomy have been associated with increased iCCA risk (+62% and +100%, respectively) in non-menopausal women compared with women 50-54 years old at natural menopause, although no association was detected with age at natural menopause [27]. Due to the different role of estrogens in HCC and iCCA female patients, it is necessary to design adequate and specific therapeutical strategies against these types of primary liver cancer both in young and aging women.

Southeastern Asia and particularly Thailand present high incidence rates of iCCA mainly due to liver fluke infection [28], however, there is no available information regarding whether this or other risk factors affect young and older people differently.

The study of different comorbidities depending on the age and gender in patients with HCC revealed that, as could be predicted, elderly patients suffered from more comorbidities [29]. Cirrhosis was the most common condition both in young and older patients, followed by HBV in patients aged <70 years and HCV in patients ≥70 years. The older group of HCC patients also showed a higher proportion of chronic diseases such as hypertension, diabetes mellitus, coronary disease and cerebral infarction and a worse quality of life.

In elders, HCC has lower accompanying fibrosis than in younger patients and it is usually diagnosed as single nodules and of larger size, which has been associated with lack of surveillance in patients without risk factors [30]. Moreover, HCC nodules are more frequently well-differentiated, encapsulated, and without vascular invasion [31].

## Hallmarks of aging and hepatocarcinogenesis

**Figure 1:**
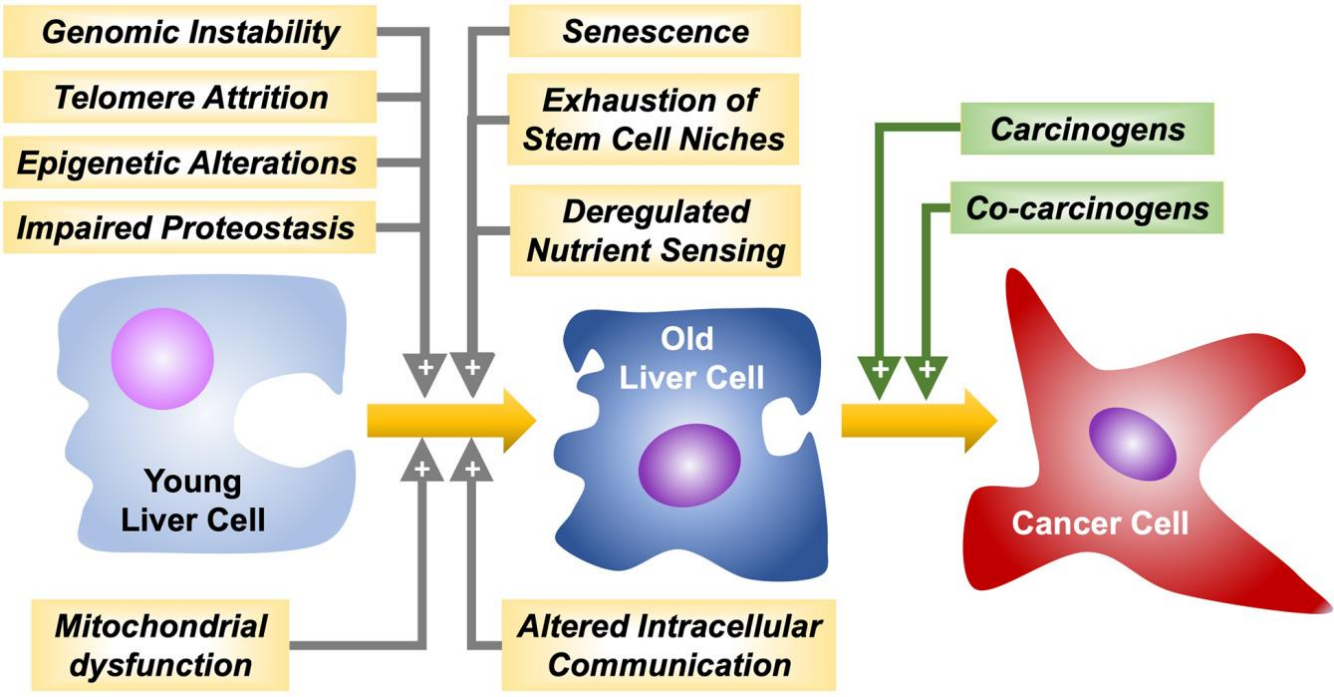
Hallmarks of aging favoring liver cancer cells malignant transformation and progression by carcinogens and co-carcinogens, respectively.

Hepatocarcinogenesis comprises a multistep process resulting in the malignant transformation of liver cells followed by tumor progression. Several common and critical cellular features considered “hallmarks of aging”, such as genomic instability, telomere attrition, epigenetic alterations, impairment of proteostasis, mitochondrial dysfunction, cellular senescence, exhaustion of stem cell niches, altered intracellular communication, and deregulated nutrient sensing [32] may play a crucial role in age-related hepatocarcinogenesis (Figure 1).

## Genomic instability

Liver fibrosis and its end-stage liver disease, cirrhosis, typically show persistent hepatocyte death and compensatory regeneration, chronic inflammation, and increased production of reactive oxygen species (ROS) [33]. All together, these features collaboratively create a pro-oncogenic microenvironment through induction of genetic alterations and chromosomal instability and by activating several oncogenic signaling pathways [34]. Genes involved in HCC pathogenesis have been classified into four major groups: i) genes regulating DNA damage response (e.g., p53); ii) genes involved in cell cycle control (e.g., RB1, p16INK4A, and cyclin D); iii) genes involved in growth inhibition and apoptosis (e.g., *M6P/IGF2R, SMAD2*, and *SMAD4*); and iv) genes responsible for cell-cell interaction and signal transduction (e.g., APC, ß-catenin, and E-cadherin) [35].

The p53 tumor-suppressor gene responds to diverse stress signals by orchestrating specific cellular responses, including transient cell cycle arrest, cellular senescence, and apoptosis. Recent studies highlight emerging roles for p53 in modulating other cellular processes, including metabolism, stem cell maintenance, invasion, and metastasis [36]. Mutations altering p53 function, together with other cooperating events, might serve to drive alterations in the cell cycle as major defects in HCC. The most frequent mutation in the *TP53* gene consists in a single base substitution, which results in the substitution of arginine for serine (p53-R249S). This represents the predominant hotspot mutation identified in 34% of all detected mutations in HCCs and is the most frequent mutation (96%) of these found in high-risk regions [37]. No significant association between the presence of *TP53* mutations and age, gender, AFP level, Child-Pugh grade, tumor size, or TNM stage has been found [37].

One primary driver of HCC is the Wnt/β-catenin signaling pathway. Mutations targeting its components are frequent in HCC (15-33%). Activating mutations in the *CTNNB1* gene, which encodes for β-catenin, are widespread in patients with well-differentiated tumors and are increased in elderly people [38]. *CTNNB1* mutations in HCC significantly co-exist with other genetic aberrant changes, such as overexpression of *MET* and *MYC* and mutations in *TERT* promoter, as well as in *NFE2L2/KEAP1, APOB*, and *ARID2* genes. Inactivating mutations or deletions are also frequently identified in *AXIN1* (10% of HCC), and more rarely in *APC* (1–2% of HCC) and *ZNRF3* (3% of HCC), resulting in activation of the Wnt/β-catenin pathway [39]. Despite the early occurrence of mutations targeting Wnt signaling components, membrane localization of β-catenin has been described as a dominant feature of HCC until advanced stages of the disease. At the plasma membrane, β-catenin interacts with multiple cadherin family members to enhance the signaling of growth factor receptors such as the epidermal growth factor receptor (EGFR). In the context of HCC, adherent junction complex disruption impairs EGFR stability to promote and support HCC cell survival. However, EGFR inhibition is not always detrimental for tumor progression as the significant level of acute tumor cell death associated with EGFR inhibition induces compensatory HCC proliferation [40]. This paradoxical mechanism of tumor progression upon β-catenin deficiency has been partly elucidated by establishing the connection between the adherent junction complex and EGFR signaling in HCC [41]. Moreover, patients with HCC harboring deficient levels of β-catenin or enhanced mutations show high rates of genomic instability, as detected by their higher frequency of loss of heterozygosity [42]. These findings suggest that abrogation of the Wnt signaling pathway could represent a divergent route to hepatocarcinogenesis. Interestingly, during aging there is a progressive deterioration in the control of the Wnt/β-catenin pathway affecting liver homeostasis [43].

Chromosomal instability emerges at an early stage during hepatocarcinogenesis, resulting in the acquisition of a malignant phenotype. Using complementary techniques, frequent loss and gain of chromosomal loci in HCC have been identified [44–47]. The loss of heterozygosity is an essential mechanism for the inactivation of tumor suppressor genes [48]. Allelic loss on 8p have been observed in high-grade dysplastic nodules, indicating that these deletions might occur in the early stage of hepatocarcinogenesis [49]. A high frequency of loss of heterozygosity and deletions of alleles on 8p22-p23 have been associated with metastasis and poor prognosis in HCC patients [50]. Besides, loss of 4q has been more frequently found in poorly differentiated HCC [51], which suggests that the inactivation of tumor suppressor genes on chromosome 4q, such as *ING2* (located at 4q34.3-35) [52], might be an important event that occurs during HCC progression after malignant transformation.

One particular characteristic of hepatocytes is the polyploid nature of many of these cells [53]. Changes in hepatocyte ploidy occur during liver injury and regeneration. Hepatocyte ploidy increases both with the aging process and in chronic diseases where proliferation is induced to compensate for ongoing loss of liver tissue [54]. Polyploidy is also a common feature in tumorigenesis, found in more than one third of human cancers [53]. However, in contrast to other tissues, in the liver polyploidy appears to protect from tumorigenesis. Thus, liver tumors arise mostly from poorly polyploid, mostly diploid, hepatocytes. In different carcinogen-driven models, higher polyploidy reduces the likelihood of HCC development, which has been attributed to the increased copy numbers of tumor suppressor genes, such as p53, in polyploid cells [55].

## Telomere attrition

Telomeres shorten during aging due to the end replication inefficiency of DNA polymerase, which accounts for incomplete DNA replication [56]. Nevertheless, mechanisms for telomere maintenance exist, such as transcriptional activation of telomerase, a telomere reverse transcriptase. This holoenzyme consists of two essential components, telomerase RNA (TERC) and telomere reverse transcriptase (TERT), which is the catalytic component undertaking synthesis of telomeric sequences using TERC as a template. TERC is constitutively expressed in normal somatic cells, whereas TERT expression is epigenetically suppressed and acts as a limiting factor for telomerase activity in most human cells [57]. However, 85-95% of all cancer cells have enhanced telomerase activity due to TERT up-regulation [58, 59].

The telomere hypothesis of cellular aging suggests that during senescence telomeres reach a critically short length and lose capping function [60]. This provides a rational explanation for the limited regenerative reserve of liver cells at the senescent stage. Besides, short telomeres correlate with the development of cancer and its malignant progression [61]. Indeed, most cancers exhibit shorter telomeres compared to the surrounding non-cancerous tissue [62, 63]. The appearance of mutations affecting the *TERT* promoter has been identified as an early event, and as the most frequent somatic mutation in HCC found in 60% of cases arising either from cirrhotic or normal liver [64]. Mutations affecting *TERT* promoter and other classical driver genes, such as *TP53* (cell cycle), *CTNNB1, AXIN1* (Wnt signaling), *ARID1A, ARID2* (chromatin remodeling), *RPS6KA3, NFE2L2, KRAS, PIK3CA, CDKN2A, CCND1/FGF19,* and *VEGFA* are found only in HCC and not in dysplastic nodules [65, 66]. All these genes play a crucial role in a cooperative or mutually exclusive manner in regulating *TERT* expression and telomere length [67]. Nevertheless, alternative mechanisms have been suggested [68]. Recent studies have demonstrated that aging, liver fibrosis, male gender, and excessive alcohol consumption are independent determinants of liver telomere attrition, which is associated with specific clinical and molecular features of HCC [69]. Dysregulated signaling pathways and the role of various mutations in telomere shortening and reactivation of telomerase during carcinogenesis both in HCC and iCCA have been recently reviewed [70].

## Epigenetic alterations

Enzymes with “writer” (DNA methyltransferases, histone acetylases, and histone methyltransferases) and “eraser” (DNA-demethylases, histone deacetylases, and histone-demethylases) function are responsible for transferring or removing chemical groups to or from DNA and histones. On the other hand, methyl-CpG binding domain proteins and other binding proteins act as “readers” recognizing methyl-CpGs and modified histones. These epigenetic events change the expression of genes involved in aging and liver carcinogenesis [71].

DNA methylation (mainly involving CpG islands in the promoter region of genes), together with hypoacetylated and hypermethylated histones, accounts for gene silencing and has been considered a biomarker of human aging rate [72]. Whereas methylation of CpGs in promoter regions has been associated with the repression of tumor suppressor genes, the same process occurring within gene bodies has been linked to oncogene induction in tumors [73]. DNA methylation is also globally altered in HCC, and aberrant modifications are associated with poorer prognosis [74]. As commented above, mutations in *CTNNB1* are frequent in HCC, and this oncogene was recently described as a key modulator in DNA methylation by increasing CpGs hypermethylation rate during aging (+0.32% per year on average) [38]. Moreover, methylation in the promoter region of tumor suppressor genes is crucial during the early stages of carcinogenesis. It has been demonstrated that oxidative stress alters the chromatin status, which leads to abnormal methylation of promoters in tumor suppressor genes, hence contributing to hepatocarcinogenesis [75]. As these alterations are potentially reversible, epigenome-targeted therapy has become a promising strategy for the treatment of cancer [76].

Post-translational histone modifications affect tumorigenesis by modulating chromatin plasticity, genomic instability, cellular senescence, and triggering the expression of genes involved in pathways promoting carcinogenesis [77]. The interplay between large histone variants and the epigenetic alterations that characterize HCC onset has been identified in HCC cell lines. Thus, protein levels of both variants of macroH2A1 (macroH2A1.1 and macroH2A1.2), an isoform of histone H2A, are increased in the livers of elderly rodents and humans and are robust immunohistochemical markers of human cirrhosis and HCC. In response to the chemotherapeutic and DNA-demethylating agent 5-aza-deoxycytidine, transgenic expression of macroH2A1 isoforms in HCC cell lines prevented the emergence of a senescent-like phenotype and induced synergistic global DNA hypomethylation [78].

Chromatin remodeling complexes are also frequently altered in HCC. These alterations include mutations in the *BRG1*-associated factors (*BAFs*) and polybromo-associated BAF (*PBAF*) chromatin complex, specifically in AT-rich interaction domain 1A (*ARID1A*; 4–17% of cases) and in *ARID2* (3–18% of cases) [38]. Histone methyltransferase *SETDB1* overexpression in HCC promotes cancer cell growth via p53 methylation and is associated with tumor aggressiveness and a poor prognosis [79].

## Impaired proteostasis

Protein homeostasis or proteostasis involves mechanisms for the stabilization of correctly folded proteins and mechanisms for the degradation of proteins by the proteasome and the lysosomes that are affected during aging [80, 81]. A conserved feature of aging across tissues, which is a crucial component of the proteostasis network, is defective autophagy. In the liver, this event is secondary to defects in intracellular trafficking of lysosomes [82].

Proteostasis and redox homeostasis constitute interconnected branches of cellular metabolism [83]. Aging is associated with perturbed stress response and repair pathways that gradually decline. The result is increased oxidative stress that induces DNA damage, disruption of proteostasis, and altered mitochondrial function [84]. Proteomic and metabolomic profiling are essential methods to enable the characterization of the system-wide molecular changes during aging and hepatocarcinogenesis. Moreover, both HCC and its treatment induce changes in liver cell proteostasis. For instance, sorafenib inhibits mRNA translation, which might constitute an adaptive stress response in HCC cells, because it protects cancer cells from ferroptosis, a form of oxidative necrosis [83].

Chaperone-mediated autophagy (CMA) is a cellular process that contributes to protein quality control. Through this mechanism, a subset of cytosolic proteins is recognized by the chaperone hsc70 that delivers them one-by-one through LAMP-2A to lysosomes for their degradation [85]. In *LAMP-2A* knockout mice, the gradual decline in protein quality control during aging reduces stress resistance and alters metabolic homeostasis, contributing to hepatocyte dysfunction and favoring malignant transformation [86].

## Mitochondrial dysfunction

Aging-associated mitochondrial dysfunction is accompanied by increased ROS, which in turn causes further mitochondrial deterioration and global cellular damage. This has detrimental effects on hepatocyte bioenergetics leading to oxidative stress, endoplasmic reticulum stress, inflammation, and cell death. Thus, in the progression from NASH to HCC, metabolic stress results in incomplete β-oxidation, impaired ketogenesis, reduced mitochondria respiratory chain activity, and ATP production, coupled with overactive tricarboxylic acid cycle. These metabolic changes favor DNA damage, the appearance of mutations, which together with the escape from cell cycle checkpoints results in enhanced risk of carcinogenesis [87].

Caspase-2 has both apoptotic and non-apoptotic functions in stress response pathways, maintaining genomic integrity, tumor suppression and aging. Progressive impaired function of this caspase is involved in age-related metabolic reprogramming, mitochondria function, and the early progression of aging [88]. In mice, the loss of caspase-2 function in older animals accelerates age-dependent alterations in mitochondrial ROS production [89]. Moreover, caspase-2-deficient mice are more susceptible to genomic instability due to their hampered ability to respond to DNA damage. Consequently, under oncogenic stress induced by diethylnitrosamine, their liver contains more damaged cells resulting in accelerated tumorigenesis [90].

## Other hallmarks of aging

The escape of hepatocytes from the senescent state is considered one primary mechanism involved in HCC development [91]. Other hallmarks of aging, such as exhaustion of stem cell niches, altered intracellular communication, and deregulated nutrient sensing, can also play a role in liver carcinogenesis. Thus, since hepatocytes play a central role in regulating the systemic response to nutrition, age-related changes in the nutrient-sensing pathways in the liver, such as insulin/IGF-1, mTOR, and sirtuins have been reported to contribute to HCC development [92, 93].

## Considerations regarding the treatment of elderly patients

Several years ago, elderly patients with liver tumors received more conservative treatments than younger patients, and, consequently, they had poorer survival [94]. However, it has been more recently accepted that the overall management strategy in the elder should not be different from that of younger patients [95]. Numerous studies have investigated the feasibility and safety of other curative (Table 2) and palliative (Table 3) therapeutic approaches in elderly patients with liver tumors, and all of them agree that advanced age itself is not a contraindication for specific treatments.

**Table 2:** Comparison of the response to curative treatments in the elderly and young patients with hepatocellular carcinoma (HCC) or intrahepatic cholangiocarcinoma (iCCA).

**Treatment**	**Tumor**	**Findings**	**Ref.**
Liver resection	HCC	Comparable effectiveness and safety Longer hospitalization and rehabilitation	[98–100]
	iCCA	Low mortality Severe complications when ≤45% remnant liver	[101]
Liver transplant	HCC	Acceptable long-term survival Controversial results in high-risk patients	[103, 104, 107]
	iCCA	Similar 5-year survival when tumor size ≤2 cm	[108]
Radiofrequency ablation	HCC	Comparable effectiveness and safety	[109, 110]
	iCCA	Comparable effectiveness in iCCA	[111]
Microwave ablation	HCC	Comparable effectiveness and safety in patients >65	[112]
	iCCA	Good survival and safety in iCCA patients when tumor size ≤2 cm	[113]

**Table 3:** Comparison of the response to transarterial chemoembolization (TACE) and systemic pharmacological treatment in the elderly *vs.* young patients with hepatocellular carcinoma (HCC) or intrahepatic cholangiocarcinoma (iCCA).

**Tumor**	**Treatment**	**Response**	**Ref.**
HCC	Sorafenib	Comparable survival benefits and safety	[120, 121]
	Lenvatinib	Comparable survival benefits and safety	[122]
	Regorafenib	Similar survival benefit in HCC patients progressing on sorafenib treatment	[124]
	Cabozantinib	Similar OS, PFS and middle-term outcome	[124, 126]
	Ramucirumab	Similar OS and safety	[127]
	Nivolumab	Similar OS	[128]
	Pembrolizumab	Similar OS and PFS	[129]
	Atezolizumab plus Bevacizumab	Similar OS, PFS and tolerability	[130]
	TACE	Good efficacy and tolerance even in >85	[114, 115]
iCCA	Gem/Cis	Similar OS	[133]
	Capecitabine	Similar effects when used after surgery	[134]
	Lenvatinib	Similar safety	[135]
	Pemigatinib	Better PFS	[136]
	Ivosidenib	Similar drug disposition	[137]
	TACE	Good efficacy and tolerance	[117]

Gem/Cis, Gemcitabine/Cisplatin; OS, overall survival; PFS, progression-free survival; TACE, trans-arterial chemoembolization.

## Curative treatments

Surgical resection of the tumor is the treatment of choice in liver cancer patients diagnosed at an early stage without cirrhosis. In contrast, transplantation can be an option for cases with cirrhosis or with advanced cancer stage [96, 97]. Advances in surgical techniques and patient care have reduced morbidity while extending survival after major liver resection. The number of elderly patients who have undergone this type of surgery has increased in recent years. Most studies conclude that liver resection can be performed in selected patients aged over 70 years as safely as in younger patients [98], and that even repeat hepatectomy may be justified for recurrent cases of liver cancer [99]. In a retrospective study on 121 curative repeat hepatectomies, elderly patients displayed more comorbid conditions pre-operatively, including hypertension and cardiovascular diseases, than the younger group; however, there was no significant difference in the incidence of postoperative complications, or in the duration of postoperative hospital stay [99]. Major hepatectomy is considered safe in elderly patients with HCC, even with cirrhosis [100]. Similar criteria apply to patients with biliary tract cancer, in whom severe complications have only been reported when the remnant liver volume was lower than 45% [101].

The fact that elderly people are at higher risk of developing complications due to more frequent comorbidities justifies that a few years ago, a liver transplant was usually not offered to HCC patients above 60-65 years old. However, the average age of liver transplant recipients has been elevated more recently [102]. Moreover, since this potential curative option has been extended to patients with CCA or liver metastasis, and antiviral agents are delaying cirrhosis development in patients with chronic hepatitis B or C, the number of elderly patients requiring liver transplant is predicted to be continuously growing in the next future. Several studies have compared the outcomes in patients aged more than 60 years or younger after orthotopic liver transplantation with controversial results; some reported lower survival rates, especially in high-risk patients [103, 104], while others found no significant differences in mortality rates [105, 106]. Acceptable long-term survival after liver transplantation has been reported in selected HCC patients older than 75 years [107]. Unfortunately, the situation is different for patients with iCCA. This is still a contraindication for liver transplantation in many centers. However, a retrospective cohort multicenter study on 29 cirrhotic patients with very early iCCA, reported a satisfactory 5-year survival (73%) independently of age [108].

Radiofrequency ablation (RFA) uses an electrical current to induce coagulative necrosis following thermal damage of tumor tissue. This treatment is considered as effective in elderly HCC patients (≥70 years) as in younger patients [109] and provides acceptable 5-year survival rates in patients older than 75 years with good performance status. Of note, those patients with comorbidities frequently die from causes unrelated to HCC [110]. Regarding iCCA, RFA is considered effective when tumor size is <20 mm independently of the patient’s age [111].

Percutaneous microwave ablation therapy is a less invasive procedure than RFA to induce tumor damage by thermal effect. Only one study has described that this treatment is safe and effective for HCC patients ≥65 years and that clinical outcome is not affected by age or comorbidities [112]. Besides, this procedure has provided an excellent long-term outcome in patients with small (≤2 cm) iCCA tumors, either under or over 65 years of age [113].

## Palliative treatments

Trans-arterial chemoembolization (TACE) is the most frequently used therapeutic approach for patients with inoperable HCC. It has been demonstrated that TACE reaches satisfactory efficacy and is well tolerated in elderly patients, including those above 85 years old [114, 115]. Recent studies have described that drug-eluting bead-TACE therapy was safe and effective in elderly patients either with HCC [116] or iCCA [117].

Regarding pharmacological treatments, although there are no specific guidelines to treat elderly patients with liver tumors and the results of clinical trials cannot always be directly translated to the general population because the participants are selected as well-fit, which does not represent what is found in clinical practice, in general, elderly patients can benefit from all the available pharmacological treatments [118]. Nevertheless, the current pharmacological armamentarium used in systemic treatments against HCC and CCA is scarcely effective and only provides modest benefits, even in young patients. Thus, tyrosine kinase inhibitor (TKI) sorafenib has been the standard of care for advanced HCC for several years [119], despite the moderate beneficial effects and some serious adverse events in some patients. It was demonstrated that the survival benefits and the safety of sorafenib were comparable in elderly and young patients with advanced HCC [120, 121]. Another TKI, lenvatinib, is now a new therapeutic option as first-line therapy for patients with unresectable HCC, and the available data indicate that it can be used safely and efficaciously regardless of age [122]. Although there are no specific studies of the efficacy and safety of other TKIs used as second-line treatment in elders, and these patients are usually underrepresented in oncological clinical trials, the available information of subgroup analyses of regorafenib and cabozantinib is promising [123]. Regorafenib provided a survival benefit in HCC patients progressing on sorafenib treatment without differences in groups older and younger than 65 years old [124]. The randomized, double-blind, phase III trial evaluating cabozantinib *vs*. placebo in previously treated patients with advanced HCC found no differences in the analysis of overall survival (OS) and progression-free survival (PFS) in patients aged <65 years *vs*. ≥65 years [125]. In another study comparing HCC patients aged ≥70 years with younger individuals, a similar favorable middle-term outcome was obtained in both groups [126].

A recent study in patients with HCC and elevated AFP after sorafenib treatment has reported that ramucirumab, a monoclonal antibody that inhibits endothelial growth factor receptor 2 (VEGFR2), showed similar OS and safety across age subgroups, including ≥75 years old, which supports its use regardless of patient’s age [127]. Regarding immune checkpoint inhibitors, the phase III CheckMate 459 trial comparing nivolumab (the first recombinant human IgG4 monoclonal antibody anti-PD-1) with sorafenib showed that OS was better in the nivolumab arm both in elderly (≥65 years) and younger (<65 years) patients [128]. Similar results were observed with pembrolizumab, another anti-PD-1 recombinant human IgG4 monoclonal antibody [129]. The phase III open-label study of patients with locally advanced or metastatic and/or unresectable HCC comparing atezolizumab plus bevacizumab with sorafenib also showed an increase in OS and PFS together with delayed deterioration of patient quality of life in the first arm both in patients under and over 65 years [130].

It was described that biliary cancer patients aged 75 years or older tolerated standard full-dose chemotherapy with gemcitabine, and the outcomes were like those seen in younger patients [131]. More recently, it has been corroborated that the patients >80 years old with biliary malignancies, when carefully selected, can potentially undergo systemic anticancer therapy and obtain a similar benefit as younger patients [132]. Moreover, survival after gemcitabine plus cisplatin, the conventional first-line treatment for advanced CCA, has been found similar in patients with advanced age (≥70 years) and younger [133]. Besides, capecitabine showed comparable effects when used as adjuvant chemotherapy following surgery in patients with resected biliary tract cancer either under or over 60 years old [134]. The still scarce results of the response to lenvatinib monotherapy as second-line treatment in unresectable biliary tract cancer have shown antitumor activity with a tolerable safety profile, with similar adverse events in patients under and over 65 years [135].

Data on FGFR targeted therapies in elderly patients with CCA are scarce, although subgroup analyses in some clinical trials are available and the results are interesting. Thus, the multicenter, open-label, single-arm, multicohort, phase II study FIGHT-202 investigated pemigatinib in previously treated, locally advanced or metastatic CCA and included in cohort A patients with FGFR2 fusions or rearrangements mainly under 65 years, in fact, 23.4% were ≥65 years (25/107) and only 5 of these patients were ≥75 years. Median PFS was higher in patients ≥75 years than in those younger than 65 years, with intermediate values in patients between 65-75 years; however, the small number of older patients means that these data should be viewed with caution [136].

Ivosidenib is a potent inhibitor of mutant isocitrate dehydrogenase 1 (mIDH1). Although no studies comparing safety and activity of ivosidenib in younger and older patients have been yet performed, a phase I study showed no differences between ivosidenib pharmacokinetics and pharmacodynamics and age in patients with adequate renal/hepatic function [137].

Despite CCA mainly affects aged subjects, this population is severely underrepresented in clinical trials, which hopefully will change in the future based on available data [138]. Fortunately, the management of advanced liver cancer is changing rapidly with new options based on different kinase inhibitors and monoclonal antibodies targeting angiogenesis that have emerged, as well as novel immune checkpoint inhibitors. Thus, recent clinical trials have recruited older patients with no maximum age exclusion criteria, and age has not been found to be predictive for treatment effect in subgroup analyses [118].

## CONCLUSIONS

Aging is a dynamic process associated with a progressive reduction in the capability of all physiological functions. The liver has a remarkable regenerative potential, so the impact of aging is somewhat less relevant than in other organs, however, over time, accumulated deterioration leads to the appearance of a senescence phenotype. Our understanding of the molecular determinants involved in the characteristics of aging, as well as their interaction with the risk factors that predispose to the development of liver cancer is still incomplete. The aging of the population worldwide, together with an increased frequency of exposure to risk factors associated with the development of these tumors, such as NAFLD and obesity, suggest that in the coming years the incidence of liver tumors in the elderly will continue to increase. Fortunately, most studies to date support the concept that, in general, all available treatments can also be recommended for elderly patients, although the comorbidities that these individuals often present must be taken into account to tailor treatment to each case and, in addition, these patients should be closely monitored.
